# Supplementary material for: Layer V Neocortical neurons from individuals With drug-resistant epilepsy show multiple synaptic alterations but lack somatic hyperexcitability
Source: PLoS One. 2026 Jan 27;21(1):e0320711. doi: 10.1371/journal.pone.0320711 (PMC12843513; doi:10.1371/journal.pone.0320711)
Supplement: Attachment — Submitted filename: Responses to Reviewers.docx [file pone.0320711.s002.docx]

**RESPONSES FOR REVIEWERS
Reviewer #1**

 Marquez et al. examined the membrane and synaptic characteristics of a few pyramidal neurons that were recorded from tissue taken from patients with epilepsy after the suspected seizure origin was surgically removed. According to the results, the primary change that can account for the tissue’s hyperexcitability occurs at the synapse level rather than the somatic level.
Given the lack of information on the electrophysiology of neurons from the human brain, especially in diseased situations, the study may be relevant. However, the manuscript requires a significant change before it is ready for publication.

**Major:**
The location of the tissue of origin must be specified, if it can be found in the surgical data. For instance, whether the tissue is hippocampus-derived greatly alters a placement in the temporal lobe. The extent to which the tissue is solely inside the seizure onset zone or includes adjacent tissue that is likely less impacted by the clinical history must also be determined. In this context, the available surgical outcome must also be described in the table of affected individuals. This aids in determining the actual contribution of the excised tissue to each patient’s network hyperexcitability.

First, we would like to acknowledge the time and effort made by the reviewer in reading our manuscript. We have taken into consideration all his/her comments and we have integrated them in both this response letter and the new version of the manuscript.

We understand the relevance of the tissue’s origin and how this factor may influence the electrophysiological properties of the recorded neurons. The cortical tissue included in our study was obtained from patients diagnosed with drug-resistant epilepsy who underwent surgery to resect the brain tissue identified as the epileptic focus. First, the epileptic foci were determined using intraoperative electrocorticography. This gold-standard method identifies epileptiform activity and guides the extent of pathological tissue to be resected. The tissue used in our electrophysiological recordings was derived from these resections, and we assume that most of our samples originated directly from the epileptic focus.

We are sorry to say that the exact anatomical location of each tissue sample is unknown, as the resected material was subsequently sectioned for different studies, including histopathological analyses, biochemical measurements, and electrophysiological analyses. Due to the loss of precise localization of the tissue used for the electrophysiological recordings, we present the results by cortical area type. On the other hand, based on the intraoperative ECoG findings by the surgical team, we understand that most of the resected tissue was pathological. For obvious ethical reasons, it is not possible to obtain healthy, non-pathological tissue for comparative purposes.

Regarding the temporal lobe samples, while most cases of drug-resistant temporal lobe epilepsy affect the hippocampus, the samples in this study were obtained from non-hippocampal regions of the temporal lobe. Samples of that nature were collected and analyzed for different studies that are beyond the scope of this study. It is, therefore, likely that the epileptic foci in these cases were in other areas, such as the entorhinal or post-rhinal cortices.

Nonetheless, in the updated version of our manuscript, we have clarified in the methods section that the examined tissue was obtained from epileptic foci identified by intraoperative electrocorticography, an experimental technique that confirms their hyperexcitable state at the network level. We hope that the reviewer understands that we cannot provide details regarding the anatomical location of the brain tissue, as the resected tissue was divided in pathological studies for clinical purposes and experimental samples for biochemical and electrophysiological experiments. Despite this limitation, we believe that the quality of the electrophysiological experiments and post-hoc reconstructions provides solid experimental evidence of the activity of human neurons obtained from brain with drug-resistant epilepsy.

Following the reviewer’s comment, the amendment included in the upgraded version of the manuscript reads on page 6 as follows: *The resected brain samples were obtained directly from epileptic foci identified by intraoperative electrocorticograms, which confirm their hyperexcitable nature at the network level*.

There are too many details missing to fully comprehend the dataset’s nature. For each patient, how many slices were taken? It is necessary to provide further details on the patient from whom the (few) recorded cells originate. For instance, one patient may have had four of the six temporal lobe cells recorded, while the other two patients may have just had one cell.

Before addressing the reviewer’s comment, we would like to emphasize that the data presented in this study are derived from whole cell patch-clamp experiments performed on human tissue obtained directly from surgical procedures. In such cases, it is not possible to establish or preselect the number of cells recorded per patient or to standardize the number of cells to be included in the study. One of the main motivations for submitting this study was the exceptional quality of the electrophysiological recordings. We are aware of the comment that we have a limited number of cells, but the quality of the recordings should be taken into account, rather than focusing solely on the limited number of cells. These are human neurons, not rodent ones. Statistical analyses serve to validate or reject data; we conducted a rigorous analysis for each parameter to validate our findings. With that said, we acknowledge the reviewer’s concern, and we have addressed this issue by including a supplementary database. This database contains all raw electrophysiological measurements corresponding to each Figure, along with detailed information on the number of recorded cells or slices per patient and by cortical region. In the updated manuscript, we present the sample size as n = number of cells / number of samples from 10 patients. For instance, IB neurons, now referred to as extratelencephalic (ET) neurons, have a sample size of n = 10 cells / 4 samples from 10 patients. This indicates that 10 ET neurons were found in only 4 out of the 10 patients. The specific origin of each cell or slice is provided in the supplementary dataset.

The statistics are presented in a too careless manner. The authors must try to justify the validity of applying their tests to such small samples for each dataset. The number (n) of measurements from which each reported mean value is derived must be included, particularly when using a repeated measures approach. Even if the result is not significant, it still needs to be reported together with the relevant p-value and other information. To clearly identify which factors were taken into account between-subjects and which were evaluated within-subjects, the mixed-effects ANOVA analysis has to be more thorough.

The use of parametric statistical tests was based on the assumption of normal distribution, which was confirmed through normality testing. Although we previously stated that normality was assessed using Kolmogorov-Smirnov tests, which were used in most cases, we also performed Shapiro-Wilk tests (originally unaddressed in the methods section). Unlike other normality tests, such as D’Agostino-Pearson or Kolmogorov-Smirnov tests, the Shapiro-Wilk test is specifically suited for small sample sizes and can be used with as few as three observations. In other words, the Shapiro-Wilk test is more sensitive for detecting deviations from normality. In the revised version of the manuscript, we now clarify that the normality of the data was validated using a Shapiro-Wilk test, which is appropriate for small sample sizes. This amendment is included in the methods section where it reads: *The normality distribution of data was validated and assessed using the Shapiro-Wilk test, which is appropriate for small sample sizes with a minimum of three observations (n = ≥3).*

Regarding the number “n” of measurements, we have revised the manuscript and we have added the *number* (n) of sample sizes for each electrophysiological parameter examined. Likewise, we selected a mixed-effects ANOVA because our design included two relevant factors influencing firing rate: the cortex type (a between-subjects factor) and current injection (or within-subject factor), following the reviewer’s comment. It is important to note that multiple current levels (0 to 390 pA, 30 pA steps) were somatically injected into the cell, resulting in repeated measures. Given that in two recorded cells, the firing rate values at higher current levels (e.g, 390 pA) were missing, we used mixed-effects ANOVA. This test is well suited to handle unbalanced data and missing values in repeated-measures designs and to account for within-cell variability by including random effects. While it is expected that increasing current magnitude leads to higher firing rates, our main aim was to determine whether firing rate is significantly influenced by cortex type. Nevertheless, following the reviewer´s observation, we have now clarified in the data analysis section that cortex type and current injections correspond to between-subjects factor and within-subject factor, respectively. The revised sentence now reads as follows:

*In case of mixed-effects ANOVA, we analyzed cortex type and current injection factors as between-subjects factor and within-subject factor, respectively.*

We hope this clarification helps the reviewer understand our rationale for not including the current injection factor (within-subject factor) as a primary explanatory variable for firing rate.

From what is said in the abstract, the focus on the cell morphology results, which are based on just (!) four cells, must be reduced.

Although we believe that including these neuronal reconstructions and morphometric data was relevant due to the information they provided, we also acknowledge the limitations of our dataset, as each human neuron was electrophysiologically characterized at the single-cell level using the whole-cell patch-clamp technique. It is worth noting that we performed biocytin labeling in every cell recorded for this and other studies (from the same tissue sample), and the results were, unfortunately, inconsistent. In some cases, we successfully stained axons, somata, and dendritic fields. However, in a larger part of the post-hoc manipulations, we obtained strong somatic labeling but no dendritic fluorescence or sparse dendritic fluorescence without somatic integrity. It is not easy to contain the excitement when a human neuron, from which all the electrophysiological data were obtained, also exhibited adequate labeling for dendritic reconstructions extending over 400 μm and a consistent distribution of dendritic bifurcations. We believe that this is not a minor or everyday achievement in electrophysiology, or even more importantly, in human electrophysiology using brain samples obtained from the operating room. However, we also acknowledge that both reviewers noted the limitations of this attainment. For this reason, we temper the description and minimize its presentation in the results section. In the upgraded version of the manuscript, we limited our statements to mentioning that a subset of pyramidal cells, which were electrophysiologically characterized, were also morphologically identified.

**Reviewer #2:**

1. Is the manuscript technically sound, and do the data support the conclusions?
The manuscript presents original research on the electrophysiological and morphological properties of human Layer 5 (L5) pyramidal neurons (PCs) from different cortical regions, obtained from surgical resections of drug-resistant epilepsy (DRE) patients. The study addresses a relevant and timely topic in human neurophysiology. However, several technical and interpretative issues need to be addressed:

• Methods: Important experimental details are missing, including the osmolarity of all solutions and the rationale for using an unusual transport solution (320 mM sucrose, 1 mM EDTA, 5 mM Tris-HCl), which could affect neuronal viability and physiology due for example to a poor ion balance given by the absence of most of the ions needed to mimic the CSF and so maintain physiological conditions.

First, we would like to acknowledge and thank the reviewer for all his/her valuable comments. Each point raised was carefully considered and thoroughly discussed, and we have made a series of amendments following the constructive criticism. We hope that the revised version of the manuscript meets the expectations outlined by the reviewer.

There is a strong rationale and backup literature to justify the sucrose-based solution to maintain the brain tissue in healthy conditions after its isolation. By using 320 mM sucrose, the osmotic pressure exerted by extracellular sodium is maintained, preventing tissue damage due to the increased excitotoxicity triggered by action potential generation or propagation. This manipulation also decreases Ca^2+^ influx and the subsequent process of neurotransmitter release; a simple method to reduce neuronal excitability. On the other hand, EDTA serves as a Ca^2+^ chelator and, indeed, a non-specific inactivator of all types of Ca^2+^-dependent enzymes that may contribute to apoptosis and cell death following the multiple traumatic events experienced by the tissue: surgery, extraction, mechanical manipulation, and then, slicing. Lastly, Tris-HCl adjusted at an adequate pH works as a buffer that resists pH changes within the biological range and preserves the tissue for experimentation. The sucrose-based solution has been used in neurophysiology for over 70 years, as first reported by Gray and Whittaker (Journal of Anatomy, 1962 Jan; 96(Pt 1):79-88) for the isolation of nerve endings. Over the years, mild modifications have been incorporated into this solution to optimize the acute brain slice process. Current literature is packed with examples of those modifications in which the current sucrose concentration, pH control, and calcium chelation are always present.

Now, regarding the possibility of abnormal neuronal physiology due to this solution, it is an unlikely scenario, as neurophysiology has been performed in acute slices obtained with sucrose-based solutions for at least 50 years, and milestone advances, including multiple forms of synaptic plasticity and intrinsic excitability, have been characterized in slices obtained in sucrose-based cutting solutions. In line with this comment, the visual quality of the electrophysiological findings included in this study does not show signs of low-quality recordings, damaged cells, or any suspicious activity of a dying neuron. By visually inspecting the firing discharge and the amplitude of the action potentials obtained during the prolonged depolarization applied in our experiments, it is noticeable that the cell’s physiological response is in good shape.

Therefore, we used a sucrose solution to preserve the human tissue and minimize damage during transportation from the operating room to the laboratory. After slicing the human tissue, the slice’s recovery was performed in a modified ACSF (details in the Methods) that included physiological concentrations of NaCl, KCl, and CaCl_2_ to facilitate physiological activation. Furthermore, the experiments were performed in standard ACSF maintained at near-physiological temperatures and with constant exposure to carbogen. This strategy, together with the quality of the electrophysiological responses obtained in this and prior (Biomedicines. 2023 Dec 7;11(12):3237.) or ongoing studies under review (Frontiers in Pharmacology, 2025; ID:1627465.), supports the notion that our experiments capture neuronal activity that closely approximates the physiological activation of human neurons.

The unusually low biocytin concentration (0.04%) is concerning, as it may limit dendritic and axonal filling. The methods section does not specify the number of cells analyzed for dendritic complexity, nor the criteria used for their selection. However, based on the results, it appears that only four cells were included in this analysis. This raises concerns regarding the representativeness and robustness of the findings, as such a small sample size limits the ability to draw meaningful conclusions.

R: We apologize for this typo. In this study, as well as in previous publications (Griego et al., 2021 (DOI: 10.1016/j.neuropharm.2020.108379), 2022a (DOI: 10.1111/bph.15735), Marquez et al., 2023 (DOI: 10.1002/hipo.23524), we have systematically reconstructed neurons with 0.4% biocytin. Thus, the mistake has been corrected in the new version of the manuscript.

Regarding the limited number of reconstructed cells, we acknowledge the reviewer’s concern. First, it is important to note that no conclusions stem from the morphometric analysis. We believe that including the morphometric data is justified, given the valuable information they provide. It is important to emphasize that each human neuron included in this study was electrophysiologically characterized, which is not a minor achievement. While morphometric analyses can be performed in *post-mortem* tissue, and patch-clamp recordings can sometimes be conducted independently on surgical samples, the combination of both approaches in our study represents an achievement in the study of human samples obtained from drug-resistant epilepsy. Of course, we acknowledge the achievements made in other studies, such as the work by Mohan et al., and in this case, we can only accept our limitations. Nonetheless, we want to mention that in some cases, we successfully labeled axons, somata, and dendritic fields. However, in a larger part of the post-hoc manipulations, we obtained strong somatic labeling but no dendritic fluorescence or sparse dendritic fluorescence without somatic integrity, and the few reconstructions that exhibited adequate labeling reached a dendritic area up to ≈400 μm and a consistent distribution of dendritic bifurcations. However, we also acknowledge that both reviewers noted the limitations of our morphometric analysis. For this reason, we temper the description and minimize its presentation in the results section. In the upgraded version of the manuscript, we limited our statements to mentioning a subset of pyramidal cells, which were electrophysiologically characterized and morphologically identified.

Similarly, the small sample size used for the classification of L5 PCs across the different cortices makes generalization difficult.

Although we understand that the small sample size in our experiments limits the generalizability of the results, our conclusions (not based on morphometrical analysis) are supported by statistical analyses and previous relevant studies. For instance, our whole-cell experiments show that Layer V pyramidal neurons, now identified as intratelencephalic-2 (IT-2) neurons, do not exhibit changes in resting membrane potential (RMP) or firing frequency when compared to previous studies using tissue classified as “normal” or “moderately affected.” Similarly, we did not observe spontaneous synaptic activity at RMP. These findings support the notion that, at the cellular level, pyramidal neurons are not hyperexcitable. However, a distinct scenario was observed in the synaptic recordings. Regardless of the cortical region, we found that synapses from brain tissue of patients with drug-resistant epilepsy showed a loss of short-term depression (STD), reflected as increased excitatory activity. Moreover, the magnitude of STD loss was positively correlated with the appearance of spontaneous postsynaptic potentials. Together, these results support that hyperexcitability observed in epilepsy may originate from mechanisms that operate at the synaptic level, such as dysregulation of STD.

• Data quantification: Several claims are not adequately supported by quantified data or statistical tests (e.g. lines 287, 296, 300-311). Notably, parameters such as action potential (AP) firing rates, adaptation, and afterhyperpolarization are mentioned but not systematically quantified or statistically compared.

R: We apologize for this omission. These lines aimed to describe qualitatively the characteristic electrophysiological differences among pyramidal cells based on their firing pattern. In the revised version of our manuscript, we have included the quantification of the measurements mentioned. We did not include statistical comparisons due to the limited number of cells recorded. For instance, only two neurons exhibited adaptive firing, while intrinsic burst and regular spiking were observed in 10 and 22 neurons, respectively.

Regarding line 287 about spontaneous synaptic activity, we did not include quantification because it was practically absent in all the cells (RS, intrinsic burst, and adaptive firing) recorded at the resting membrane potential.

• Neuron classification: The classification of L5 PCs based solely on spike frequency adaptation and firing rate is oversimplified. Extensive literature demonstrates the existence of well-defined subpopulations (ET vs. IT; for human temporal cortex gyrus see for example Kalmbach et al., 2021; for rodent neocortex see Moberg and Takahashi, 2022). These cells have distinct morphological and electrophysiological features that must be taken into account while attempting to classify L5 PCs in human cortex. The authors should revisit their classification strategy in light of established frameworks.
We are grateful for the constructive feedback provided in this comment. We have reviewed the mentioned works and adopted the classification used by Kalmbach et al. (2021). Interestingly, we found that our previous classification according to the firing pattern aligns precisely with that of Kalmbach et al. Specifically, pyramidal cells exhibiting burst firing correspond to extra-telencephalic (ET) neurons; those with regular spiking correspond to intratelencephalic-2 (IT-2) neurons; and those with adaptative firing correspond to the IT-1 neurons. Given the predominance of IT-2 and ET neurons in our recordings, we first compared their electrophysiological features and subsequently focused our analysis on the IT-2 population characterized by regular spiking firing. Given the limited number of IT-1 neurons (n=2), we only included them to illustrate their firing pattern in Figure 1D, as part of the overall distribution of the recorded cells. The corresponding measurements are included in the supplementary database.

Based on the electrophysiological characteristics more relevant for distinguishing these neurons, we compared their firing rate, instantaneous firing frequency, firing adaptation, somatic input resistance, and sag ratio. The latter was used as an indirect readout of functional expression of HCN channels, which are highly expressed in both ET and IT neurons but at different densities.

• Results interpretation: The interpretation of the FV-fEPSP relationship and the claim of synaptic imbalance in DRE lacks proper statistical comparison (e.g., regression slopes) and validation against healthy tissue or additional rodent data. Several passages are speculative and need either experimental evidence (e.g., recordings with synaptic blockers) or should be moved to the discussion.

Thanks for the comment. As expected, working with human brain resected from the operating room also imposes restrictions. Being one of them, the absence of healthy tissue prevents adequate comparisons. Nevertheless, these limitations can be addressed by applying mathematical analyses; in our specific case, the least squares method, to provide theoretical evidence of deviations from the expected physiological pattern (the values obtained in the rat) when compared with the responses observed in the human DRE acute slice, even in the absence of direct comparisons with healthy human tissue.

As we stated in the manuscript, previous studies performed in rodents have demonstrated that the loss of the linear correlation between FV and fEPSPs indicates synaptic imbalance or loss of synaptic strength. We also acknowledge the reviewer’s concern about speculative interpretations extracted from this analysis. Therefore, we have tempered the speculative content in the discussion section of the new version of the manuscript.

In line with the observations raised by the reviewer’s, we like to discuss the possibility of pharmacological experiments to block elements of synaptic transmission. We strongly believe that this manipulation would increase the complexity of the analysis instead of limiting the problem. The comparisons included in this study were made considering that synaptic transmission comprises inhibitory, excitatory, and even modulatory elements, which collectively shape synaptic strength and, more importantly, if the presynaptic elements are altered (in this case, the FV, but can also be NT release) the postsynaptic responses are indeed altered, which modifies their linear relationships. The predictions obtained with the square least method, both in the human tissue and the rodent example, support this tenet. Our data suggest that, similar to what is observed in the rodent’s brain, in the DRE cortical human synapse, a disbalance in the FV and the fEPSPs occurs that contributes to the synaptic alterations that occur in the human brain. The next understandable step would be to isolate the different elements that shape the synaptic strength (the pharmacological approach directed to the Glutamatergic, GABAergic, and modulatory elements of these synapses) to determine the degree and locus of alteration underlying the changes observed in the drug-resistant epilepsy brain.

• Additionally, the morphological analysis in this study raises concerns about the representativeness and robustness of the findings, as the small sample size and large neuronal heterogeneity in human L5 (e.g., Kalbach et al, 2021) limit meaningful conclusions. Reported values, such as a total dendritic length of ~100 μm and an average of 1.5 branches, are unusually low compared to published data (e.g., >10 mm dendritic length and >60 apical branches in Mohan et al., 2015; Kalbach et al., 2021). These discrepancies may result from technical issues, including low biocytin concentration (0.04%?) or tissue damage during slicing. While the authors briefly mention some limitations, they still draw conclusions as if these morphological parameters were fully representative. Given the small sample size, possible artifacts, and inconsistencies with previous literature, these findings should be interpreted with caution, and further validation with larger, methodologically optimized datasets is strongly recommended.

We understand and agree with the reviewer’s comment. First, we temper the mentions to the morphometric analyses, and we have rephrased our text to minimize the interpretation of these experiments. As previously stated, considering the relevance of having cells that were both electrophysiologically recorded and morphologically identified, we maintained the Figure while minimizing possible interpretations. Please keep in mind the limited access and information regarding reconstructions of human cells, and more importantly, from electrophysiologically characterized cells obtained from DRE patients. We know that the morphometric information extracted from our cells can be useful for anybody within the scientific community who wants to use the morphometric values of human neurons. They are not definitive values, but a small contribution that can be used for larger studies. Interestingly Mohan et al., reported that they used dendritic morphologies obtained from Neuromorpho.org; our morphometric measurements and final images will be part of the same database where we have contributed previous reconstructions. Lastly, as found in public databases such as the Allen Brain Atlas, the number of human neocortical cells characterized both electrophysiologically and morphologically remains limited. Here we have a small contribution, and we have removed any further mention to minimize interpretations.

• Figures: Several figures lack error bars, sample size annotations, and statistical comparisons. Some traces are presented without supporting quantification and should be either properly quantified or removed. Additionally, certain visualizations are unnecessarily complicated. For example, in Fig. 1B, expressing the number of cells as percentages makes the information less accessible than it needs to be, and the actual numbers should be reported directly.

We have updated the figures of the manuscript to show the data as point and line plots with standard deviation, as indicated. Likewise, we have included the necessary statistical comparisons that were previously omitted. These comparisons, along with the corresponding sample sizes, were primarily conducted for the data presented in Figure 1. As mentioned in an earlier response, we reconsidered the classification of pyramidal neurons into extratelencephalic (ET) and intratelencephalic (IT-2) types based on firing pattern, input resistance, firing frequency, instantaneous frequency, adaptation, and sag ratio—parameters that clearly differentiate ET from IT neurons. Regarding Figure 1b, we have presented the values both numerically and as percentages. These changes are reflected in the new version of Figure 1.

In Fig. 5C, D, and F, the heatmaps could be replaced with simple numeric values, as only four cells were analyzed.

We are sorry for this shameful error. The actual figure legend included in the previous version of the manuscript referred to heatmaps that were removed before submission, and, mistakenly, we did not update the figure legend. We have amended this mistake. Fig 5B shows a simple two-dimensional metric representation of the neuronal size and the dendritic/axonal spread in the X-Y axis. Regarding the numeric values included in Figures 5C and 5D: first, they are scatter graphs used to create the Sholl graphs in Fig 5F. The Sholl graphs are a standard representation of the intersections. Interestingly, the Sholl values included in our study have a similar distribution to the one reported by Mohan et al. Coincidentally, these authors represented individual Sholl values for the cells in the same way we chose to show our morphometric values.

Importantly, we followed the reviewer’s comment, and we limited our wording to mention that a subset of neurons electrophysiologically characterized were also morphologically identified.

In summary, while this study explores an important and promising area, substantial revisions are required to ensure that the conclusions are rigorously and appropriately supported by complete, quantified, and statistically tested data.

Thanks for this comment. We are aware of the limitations regarding the number of cells included in our study, particularly in relation to the morphometric data and the even smaller subset of reconstructed neurons. While it is common to report large numbers of cells in rodent studies (for electrophysiology or morphometric measurements), research on human tissue presents substantial challenges. Access to freshly resected surgical tissue is limited due to both ethical and logistical constraints, and because several groups work simultaneously on diverse questions using the same resected tissue. Nevertheless, we believe that the quality of our electrophysiological recordings represent a meaningful contribution toward understanding specific phenomena in the context of DRE. Most importantly, we hope that these data, although limited in number, will serve for future studies aimed at achieving a more comprehensive understanding of the human brain.

2. Has the statistical analysis been performed appropriately and rigorously?
Statistical analyses are generally described but are not always rigorously applied:

• Missing analyses: Many important comparisons (e.g., between cortical regions, between firing pattern types) lack proper statistical testing or explicit reporting of sample sizes.

R: We apologize for the omission of the statistical comparisons, particularly those related to Figure 1. We have incorporated the necessary statistical comparisons, along with their sample sizes. As mentioned in a previous response, our first aim was on the comparative electrophysiological analysis between the extratelencephalic (ET) and intratelencephalic (IT)-2 neurons. Therefore, all the statistical comparisons related to Figure 1 were performed using unpaired tests, as the data were normally distributed.

For example, in lines 430-438, the description of spontaneous synaptic events following train stimulation requires clarification. It is unclear whether these events represent recurrent excitation, asynchronous release, or another process.

The nature of the spontaneous synaptic potentials during the 30 Hz train remains unclear and was not addressed in the discussion, and we apologize for this. However, such spontaneous events do not occur in physiological conditions either in rodent or human neocortex (Crabtree et al., 2017; Testa-Silva et a., 2014).

In the new version of our manuscript, we have addressed in the discussion, page 27, a possible explanation of the nature of the spontaneous synaptic activity based on previous works. This sentence reads as follows:

*The appearance of spontaneous postsynaptic potentials during 30 Hz train stimulation is striking, and it may be interpreted as a consequence of a transient increase in excitatory activity due to the brief high-frequency stimulation, as previously discussed. While recurrent spontaneous activity in deep layer V of the neocortex is a well-documented phenomenon (Beltramo et al., 2013), this activity is typically not observed during periods of brief high-frequency stimulation under physiological conditions in rodents (Crabtree et al., 2017) or near-physiological conditions in humans (Testa-Silva et al., 2013). However, given that this recurrent spontaneous activity is locally controlled by a subset of layer V pyramidal neurons (Beltramo et al., 2013), the loss of STD in LI/II – V synapses, or alternatively, the emergence of synaptic facilitation (as observed in temporal cortex) during 30 Hz train stimulation, might provide the initial excitatory drive required to trigger such spontaneous activity. Although appealing, this hypothesis requires additional investigation to determine whether the loss of STD or the gain of facilitation during brief high-frequency stimulation contributes significantly to the network hyperexcitability observed in drug-resistant epilepsy.*

The claim that the number of these events “tended to be higher” in the temporal cortex lacks statistical validation.

R: We regret this oversight in statistical validation. In the revised version of our manuscript, we have now included a one-way ANOVA followed by a Tukey’s post hoc test, which detected significant differences. This change reads on pages 24 and 25 as follows:

*While the number of spontaneous postsynaptic potentials was significantly higher in temporal neocortex slices compared to parietal slices, no statistically significant difference was found when compared to frontal neocortex slices (events in temporal cortex: 9 ± 2.65; in frontal cortex: 6.67 ± 3.21; in parietal cortex: 2.75 ± 0.96; one-way ANOVA, F_(2,7)_=0.446, Tukey´s post-hoc test, p < 0.05 in temporal vs. parietal cortex; Fig 4F).*

Further analysis is needed to determine whether this facilitation reflects pathological hyperexcitability or normal synaptic dynamics.

R: As mentioned in a previous response, in the updated version of the manuscript, we mention on page 27 that additional investigation is required to determine whether the loss of STD or the gain of facilitation during brief high-frequency stimulation contributes significantly to the network hyperexcitability observed in drug-resistant epilepsy.
• Choice of error metrics: The use of the standard error of the mean (SEM) rather than standard deviation (SD) with such small sample sizes can give a misleading impression of data variability. SD would be more appropriate to accurately reflect the dispersion within the sample, particularly in cases where individual variability is of interest.

R: Thanks for this pointed observation. In the new version of our manuscript, we presented the data as mean ± standard deviation to show the data spread both in results section, as Figures and its legends.

• Linear regression analyses: The interpretation of r² values in the FV-fEPSP relationship needs to be more cautious (lines 399-406). Perfect or near-perfect r² values (such as an r² = 1 in the frontal cortex data) may indicate overfitting that might be given by some issues in the data such as limited data points. Additionally, the phrase “theoretical consistency and proportionality” is vague and should be explicitly defined how the FV-fEPSP relationship in DRE in altered by comparing it with “healthy” tissue (i.e. non-epileptic, for example access tissue from tumor resections) or more rodent data. The least squares method quantifies the strenght of a linear relationship, but it does not inherently substantiate or prove synaptic imbalance. A statistical comparison of regression slopes across conditions is needed to support this claim. Finally, the claim that the proportionality is “disrupted” in DRE should be backed by statistical evidence.

Following the reviewer’s comment, in the updated version of the manuscript, we have adopted consistent terminology and calculated and compared the regression slopes for each cortical area relative to the control group. We found significant differences between slopes, which suggest alterations in synaptic gain, likely to reflect disruptions in synaptic efficacy or network excitability, and supporting the presence of synaptic imbalance in DRE. However, the application of the least squares method reveals that this relationship is not entirely lost. Moreover, the slopes derived from the least squares analysis demonstrate recovery across all areas relative to the control group, suggesting that the fundamental linear input-output relationship and its associated synaptic gain remain preserved and consistent with basal physiological conditions despite underlying disruptions.

• Assumptions and justifications: The rationale behind choosing specific current injection ranges and classification criteria is unclear and requires clarification. For example, was the range 90-210 pA empirically chosen, or was the firing linear without any adaptation, or was there the absence of significant variability in firing rates?

R: The analysis of neuronal gain was conducted to corroborate differences in firing rates between cortices. Although we did not explicitly mention it, the range from 90 to 210 pA was chosen empirically based on the linear portion of the current-firing rate curves. This range was characterized by null or sparse adaptation, in contrast to higher current magnitudes (e.g., 270 pA). In the updated version of the manuscript on page 10, we specified in the methods section that neuronal gain was calculated as the slope of the linear fit to the rate firing–current plot using responses to current injection from 90 to 210 pA, which exhibited null or sparse firing adaptation.

3. Have the authors made all data underlying the findings in their manuscript fully available?
The data availability is generally acceptable but lacks clarity in several areas:
• The number of cells analyzed for dendritic complexity is not specified in the methods, though results suggest only four were used, raising concerns about representativeness.

R: The reviewer’s comment is correct. The morphological and dendritic complexity analyses were performed on only four cells, as now specified in the Methods section on page 17. Given the limited number of cells analyzed, we have moderated our conclusions and discussion related to these results. The data are now presented primarily as morphological confirmation of pyramidal cells that were electrophysiologically identified as regular spiking. As mentioned in a previous response, we have interpreted these findings with greater caution and reduced their emphasis in both the abstract and the conclusions.

• The availability of raw electrophysiological data (e.g., individual firing frequencies, membrane properties, and fEPSP measurements) is not explicitly confirmed.

R: We thank the reviewer for this observation. We have included a supplementary database containing all raw electrophysiological data corresponding to each Figure. This database provides individual measurements for each recorded cell (e.g., firing rate frequencies, passive membrane properties, neuronal gain, etc.) or slice recorded (e.g., fEPSP waveform parameters, paired-pulse ratio, short-term plasticity, and spontaneous postsynaptic potentials).
• Data supporting several figure panels (e.g., spontaneous postsynaptic potentials, firing rates) are either missing or insufficiently described. The authors should ensure that all underlying data points (not only summary values) are made available.

R: We apologize for this omission. The firing rates are now presented in Figure 1 and described in the corresponding text. Regarding spontaneous postsynaptic potentials, we have clarified that these events do not occur under physiological conditions in rodents or near-physiological conditions in humans, as mentioned in a previous response. The possible origin of this phenomenon is now briefly discussed in the Discussion section.

4. Is the manuscript presented in an intelligible fashion and written in standard English?
The manuscript is generally intelligible and written in acceptable English, but several issues of clarity, consistency, and terminology need to be addressed:
• Abbreviation consistency: All abbreviations must be defined at first appearance and used consistently throughout the text.

R: We appreciate this observation. We have carefully reviewed the manuscript and ensured that all abbreviations are consistently defined at their first appearance and correctly used in the text.

• Sentence clarity: Several sentences in the Introduction and Methods are overly long or ambiguous (e.g., lines 69-73 and 74-79). These should be split and clarified.

R: We have paid special attention to the sentences mentioned to improve their length and clarity. The revised sentences are as follows:

Lines 69 to 73

*These differences, along with the limited understanding of human neuronal physiology in its normal state [10,11]**, may contribute to misinterpretations of the cellular mechanisms underlying the hyperexcitability observed in epilepsy.*

Lines 74 to 79

*Remarkably, layer V PCs have been identified as a primary source of interictal discharges in epilepsy [19–21**], a phenomenon that may be partially explained by their morphological and biophysical properties [22,23].* *Therefore, this study examined the biophysical and synaptic differences of layer V PCs in the temporal, parietal, and frontal cortices resected from patients with drug-resistant epilepsy (DRE).*

• Technical terminology: Terms like “synaptic spontaneous activity” should be replaced by the more precise “spontaneous postsynaptic potentials.”

R: Thanks for this precise observation. We have replaced the term “spontaneous activity” with “spontaneous postsynaptic potentials” in the results from extracellular recordings. In the case of whole-cell recordings, we have retained the term “spontaneous synaptic activity,” as it more accurately reflects the nature of the data.

In line 162, it is not clear what the authors mean with “interval’s latency”. If I understand Fig. 1 correctly, the latency refers to the time between the onset of the current injection and the occurrence of each evoked action potential within that step - is this what the authors meant?

R: We apologize for the confusion caused by using the term “interval’s latency”. We intended to refer to the time of occurrence of the second of two successive spikes, as described by Hemond et al. (2008). The reason for using the second spike is that the first spike does not follow a prior response and therefore lacks an interspike interval, unlike the second of subsequent spike pairs evoked during the current step. Following the observation of the reviewer, in the new version of the manuscript, we have amended this misunderstanding. This clarification reads on page 10 as follows:

*The spike frequency adaptation was calculated as the slope of the linear fit of the relationship between the interspike interval (ms) and the time of occurrence (ms) of the second spike in each pair of consecutive spikes, elicited with a current pulse of 210 pA (1 s).* This clarification is also reflected in the x-axis label of the plot shown in Figure 1G.

It should be also better clarified whether the authors refer to cytoarchitectonic subdivisions of Layer 5 (e.g., 5a vs. 5b) or a particular distance from the pia when they write “the middle region of layer V” (line 277) or “external region of layer V” (line 375).

R: We apologize for this mistake. The subdivision of Layer V where the recordings were conducted corresponds to layer Va, which is located closer to the pia compared to layer 5b. This decision was made to avoid polysynaptic contamination of fEPSP or the appearance of population spikes, whose incidence increases if the recording electrode is positioned on layer Vb. In the revised version of the manuscript, we have clarified that recordings were performed on Layer Va, as indicated on pages 11 and 21.

• Consistency in tense and form: Active/passive forms should be harmonized, and descriptions of results made more precise.

• Figure labels: Some figure labels are misleading or incorrect (e.g., calling voltage traces “I-V curves” in Fig. 2B1-B3).

R: We apologize for this mistake. We have replaced “I-V curves” with “voltage traces”.
Overall, while the manuscript is understandable, careful editing for clarity, grammar, and terminology is needed.
**Summary:
Major points**

R: We have addressed the majority of the comments (now presented as a summary), both major and minor, in our previous responses. Additional clarifications were provided when deemed necessary. We appreciate the reviewer’s valuable contributions to improving the manuscript.

• Please add missing details in the Methods section, including osmolarity of solutions, rationale for transport medium, objective lenses, z-stack step size, and sample size criteria for morphology analysis.
• Reconsider the classification strategy for Layer 5 pyramidal neurons in light of established subtypes (ET/IT) as described in recent literature (e.g. Kalmbach et al., 2021; Moberg and Takahashi, 2022).
• Quantify all electrophysiological parameters discussed in the text (e.g., AP firing rates, spike adaptation, afterhyperpolarization), and report statistical analyses.
• Correct overinterpretations regarding synaptic influence on firing rates and the FV-fEPSP relationship. These claims should be either experimentally tested (e.g., with synaptic blockers) or restricted to the discussion.
• Review and improve figure presentations, i.e. add error bars, report the number of cells, perform statistical comparisons, and remove unsupported traces.
• Replace speculative interpretations with data-supported statements or move them to the discussion.
• Revise the language for clarity, consistency, and technical accuracy, addressing long or ambiguous sentences, terminology, and typographical errors.
**Minor points**
• Adjust terminology, e.g. “spontaneous postsynaptic potentials” instead of “synaptic spontaneous activity.

R: We have used the term “spontaneous postsynaptic potentials”, as appropriated.
• Clarify specific method descriptions (e.g., “Membrane capacitance was calculated as the ratio of the membrane time constant to the input resistance”).

R: This confusion has been addressed in the methods.
• Correct typographical and figure annotation errors (e.g., r² values in line 398, significance asterisks in Fig. 3C).

R: We have amended these mistakes. Thank you.
