# Supplementary material for: Layer V Neocortical neurons from individuals With drug-resistant epilepsy show multiple synaptic alterations but lack somatic hyperexcitability
Source: PLoS One. 2026 Jan 27;21(1):e0320711. doi: 10.1371/journal.pone.0320711 (PMC12843513; doi:10.1371/journal.pone.0320711)
Supplement: Attachment — Submitted filename: Responses for reviewers.docx [file pone.0320711.s003.docx]

**Responses for reviewers’ comments**

**Review 2**

I thank the authors for the thorough and thoughtful revisions to their manuscript. The new version is much improved in clarity and presentation, and several of the concerns I raised in my initial review have been well addressed. I appreciate the substantial improvements made in the revised manuscript, particularly the more careful interpretation of the data. This is a welcome change. While I fully understand the challenges involved in working with human cortical tissue – and I acknowledge the value of such data - I would still encourage the authors to frame their conclusions with appropriate caution given the sample size. The difficulty of the preparation is not in question, but ensuring careful interpretation remains important for the strength of the study.

Overall, I believe the manuscript is close to being suitable for publication pending minor revisions. I do have a few additional comments and suggestions:

1) Thank you for adding the osmolarity value of the intracellular solution (315–325 mOsm/l). While this value is somewhat high compared to typical physiological intracellular osmolarities (~280–295 mOsm), I understand it may not have impacted the current dataset substantially. However, please report the osmolarity also of the other solutions used for transport, slicing, recovery and recording (extracellular aCSF) for completeness.

R: We have added this information to the revised manuscript. The osmolarities of the solutions were approximately 315 mOsm for the transport solution, 290 mOsm for the slicing solution, 315–320 mOsm for the incubation solution, and 290–295 mOsm for the aCSF solution. These values are now shown in methods section.

Also, we followed the reviewer comments, and we included a “limitations of the study” paragraph in the discussion section of the study. We have temper our interpretations and acknowledge our limitations in the study.

2) Please include the quality criteria for inclusion of patch clamp recordings, including series resistance and the max % change of it at the end of the recording. Please also add the calculation of the liquid junction potential and if the RMP was corrected for it.

R: The series resistance values recorded ranged from 14 to 25 MΩ, and cells that exhibited a >20 % change were excluded from analysis. The liquid junction potential was 12–15mV, and the RMP values were not corrected for it. This information was added to the methods section (lines 199 to 201).

3) Please specify in the Methods section the membrane potential at which cells were held during current-clamp recordings. Additionally, indicate this value (i.e., the RMP) in the example traces in Figure 1D, both for the responses to current injections and for the spontaneous synaptic activity traces.

R: For current-clamp recordings, intrinsic membrane properties were determined at RMP, and the firing measurements were performed at a holding potential of −70 mV. This information is included in the Methods section of the revised version of the manuscript. Since representative traces by firing type and SSA traces were obtained from the same cell, we have added the RMP value on the left side of each representative trace by firing type in Figure 1D.

4) Line 423: the manuscript states that data from “a control rodent´s acute slice” were used for comparative purposes. However, it appears that the rodent dataset consists of only one slope value (n =1). If so, this does not support statistical testing, as no variance can be estimated from a single observation - even if a standard deviation (SD =5.68) is later reported. Unless multiple independent rodent data points are available, I recommend presenting this comparison descriptively, without inferential statistics. If instead this was a typo, please correct it and include also the sample size of rodent’s data in the figure legend. Moreover, since the data were taken from a publication, please cite it also in the results section.

R: Thanks for noticing this mistake. It was a typo, now corrected to “control rodent’s acute slices.” For the correlation analysis in the rodent cortex, we used measurements of 91 events (fEPSPs) obtained from 5 slices across 3 animals, which are now included in the revised version of our manuscript both results section and corresponding figure legend.

5) Line 427: minor typo in the value of r2.

R: Thanks for this observation. We had corrected the mistake.

6) Please review again the use of abbreviations for consistency. For example, the abbreviation “RS” (regular spiking) is used in the text but is not defined at first appearance (e.g., line 167). While it’s acceptable to remind the reader that IT-2 neurons are regular spiking, for clarity and consistency, please use either “IT-2 neurons” or “RS neurons/PCs” throughout. For instance, in the morphological analysis section, the term “RS PCs” appears, which may be confusing without consistent terminology.

R: We have revised the consistency of abbreviations. RS was defined at its first appearance (line 208 in revised manuscript), and RS neurons in the morphological analysis section were replaced by IT-2 neurons for consistency. Furthermore, abbreviations with low frequency, such as intrinsic burst (IB) and adaptive firing (AF), have been removed.

7) Discussion: the authors compare RMP values across studies and relate it to the age limitation (range 2–30 years). However, such comparisons are inherently limited: RMP measurements in whole-cell patch clamp are strongly influenced by recording conditions, including pipette and bath solutions, series resistance, dialysis effects, and the often-uncompensated liquid junction potential. Without standardized protocols, direct comparisons of absolute RMP values between studies are not reliable. Furthermore, the cited age range may not represent a major limitation. Barzó et al. (2025, eLife) analyzed a large dataset of human L2/3 pyramidal neurons (from cortical tissue close to pathological lesion – but in line also with non-human primate and rodent studies) across the lifespan and showed that the most pronounced changes in passive properties, including RMP, occur early in life (before age 1), with relatively stable values beyond that. This suggests that developmental shifts in RMP are unlikely to confound comparisons within the 2–30-year range, provided the data were recorded consistently.

R: We thank you for this observation. We acknowledge that RMP measurements are strongly influenced by the experimental recording conditions, as mentioned by the reviewer. We also appreciate that the reviewer brought up the work by Barzó et al. (2025), which allows us to better compare our data and refine the discussion narrative. However, we would like to highlight one observation from Barzó et al. While it is clear that the dramatic changes in passive membrane properties occur during the first year of life, subtle yet significant differences, including in RMP, can still be observed across life stages.

Although our study shows that, despite age differences (2–7 years, childhood, for the parietal cortex; 21–30 years, early adulthood, for the temporal and frontal cortices), RMP is relatively homogeneous across the regions examined, this remains an interesting observation. Barzó et al. (2025), using “non-pathological” or non-epileptic tissue, reported that RMP becomes significantly more hyperpolarized between early childhood and early adulthood (mean ± SD: –65.44 ± 6.82 mV vs. –68.69 ± 5.54 mV). While we recognize the difficulty of directly comparing RMP values across studies, the findings of Barzó et al. (2025), along with those cited in our discussion, support the notion that RMP values obtained from tissue derived from epileptic foci are more hyperpolarized than expected under non-epileptic conditions, possibly reflecting underlying pathological or compensatory mechanisms. In the revised manuscript, we have modified the paragraph in the discussion section to address this idea in the following paragraph (page 29):

*Another important consideration is that electrophysiological measurements may vary across life stages (Barzó et al., 2025). For instance, the RMP in layer II/III PCs from non-epileptic tissue becomes more hyperpolarized from childhood to early adulthood (mean: –65 mV vs. –69 mV). In our study, despite age differences between the examined cortices (2–7 years, childhood, for the parietal cortex; 21–30 years, early adulthood, for the temporal and frontal cortices), the RMP was predominantly more hyperpolarized (–72 to –73 mV) than in cortical neurons from non-epileptic tissue (Moradi Chameh et al., 2021; Barzó et al., 2025). These observations support the idea that a more hyperpolarized RMP may be an underlying feature of epileptic tissue. However, it is worth noting that other factors, such as series resistance and liquid junction potential, can also influence RMP measurements.*

8) Please, be consistent also with citation style as sometimes (Last name et al, year) is provided instead of numbers.

R: We have amended this mistake. Thanks.

**Reviewer #3:**

Review of “Layer V Neocortical Neurons From Individuals With Drug-Resistant Epilepsy Show Multiple Synaptic Alterations but Lack Somatic Hyperexcitability”

The authors performed whole-cell recordings from human layer-V pyramidal cells resected from the temporal, parietal, and frontal cortex in patients with drug-resistant epilepsy. Cells were classified as IT-2 (regular-spiking) and ET (intrinsic-bursting), with regional analyses primarily focused on IT-2 neurons. Using L I/II stimulation while recording in L Va, they measured presynaptic fiber volleys (FV) and field EPSPs. FV–fEPSP correlation was weak in human tissue but much stronger in a rodent control. A key finding is that 10-pulse, 30-Hz trains produced significant facilitation in the temporal cortex, whereas the frontal cortex showed only mild facilitation, and the parietal cortex showed none. These synaptic results were accompanied by more spontaneous postsynaptic events in the temporal cortex than in the other regions. In conclusion, the authors state that differences in synaptic properties, rather than cellular electrophysiology, underlie epilepsy.

The authors applied solid, classical methods and clearly state their aims. It is also important to enhance the human electrophysiological recording dataset, as done by the authors, rather than relying on a single dominant source of data. Nevertheless, several concerns arose during reading, some about the results themselves and others about the strength and framing of the conclusions. In some cases, the lack of proper control does not prevent publication but requires the authors to adjust their conclusion.

**Major comments**

• SSA and time from resection (Figure 4G).

SSA appears highly related to elapsed time after surgery/slicing (larger immediately post-resection). Did the authors record the time from surgery to experiment for each neuron and analyze SSA versus this interval?

R: We did not examine the relationship between the time from surgery and the timing of the analysis of spontaneous postsynaptic potentials shown in Figure 4. We would like to clarify that we measured SSA at the somatic level using whole-cell recordings (Figure 1) and spontaneous postsynaptic potentials elicited by gamma train stimulation (10 stimuli at 30 Hz) at the synaptic level using extracellular recordings (Figure 4). In both cases, the average time between brain tissue extraction during surgery and the beginning of electrophysiological recordings was 3 ± 0.5 h.

As we stated in our manuscript, the recorded neurons did not exhibit SSA at resting membrane potential in the whole-cell configuration. However, spontaneous postsynaptic potentials emerged during 30 Hz stimulation, which were highly correlated with the magnitude of facilitation in response to the 30 Hz train (Figure 4G), possibly enhancing excitatory drive during high-frequency periods of neuronal activity. Therefore, although it may seem logical that an earlier start of electrophysiological recordings would be associated with vigorous spontaneous postsynaptic activity, the consistent absence of SSA in whole-cell recordings and the controlled average delay between tissue extraction and the onset of experiments make such a relationship unlikely.

• Sag ratio (IT-2 vs ET).

Reported sag ratios (IT-2: 0.141 ± 0.072; ET: 0.164 ± 0.063) are smaller than expected from prior work and my own experience. Since rodents were also recorded, a rodent sag comparison could be informative. As it stands, the small difference raises concern that the ET vs IT-2 classification may be compromised.

R: Thanks for this observation. There are several factors that could explain this difference. First, methodological differences in measuring the sag ratio. For example, Kalmbach et al. (2021) reported sag values between 1.1 and 1.25, with ET neurons exhibiting a significantly higher sag ratio than IT-2 neurons. These values are considerably higher than our measurements. In our study, we calculated the sag ratio as the proportion between the voltage change from the maximal hyperpolarizing deflection to the steady-state voltage and the total voltage change from the resting membrane potential (RMP) to the maximal deflection in response to a −300 pA current injection, following a classical method for sag ratio estimation. Using the method described by Kalmbach et al., our measurements increased by approximately one unit, yielding values closer to those reported by Kalmbach et al.

Regarding the possible influence of sag ratio values on neuronal classification, we would like to clarify that the primary criterion for classifying neurons as ET or IT (type 1 and type 2 subtypes) was their firing pattern, specifically whether they exhibited intrinsic bursting or regular spiking. This parameter is considered the most reliable criterion for neuronal classification. That said, the absence of differences in sag ratio values observed between ET and IT-2 neurons are more likely explained by the tissue origin, which was derived from epileptic foci in patients with drug-resistant epilepsy, and by potential alterations in HCN channel expression, as briefly mentioned in the new section titled Electrophysiological Differences between IT-2 and ET neurons.

• Input resistance of ET neurons.

ET Rin of 122 ± 24.1 MΩ seems high relative to prior studies (around 50 MΩ). This calls into question the classification and could impact conclusions about IT-2 properties. Please reconcile with the literature and methods.

We understand this concern. The first criterion used to differentiate pyramidal neurons as IT-2 or ET neurons was their firing pattern—regular spiking or burst spiking, respectively. Once this classification was established, both groups displayed the classical differences in other electrophysiological parameters, such as spike adaptation, instantaneous firing frequency, and input resistance, which are among the most robust electrophysiological distinctions between these neuronal populations (Kalmbach et al., 2021). Regarding the higher input resistance values observed in our experiments compared with previous studies, several factors could contribute to these differences such as health status if the tissue (non-epileptic tissue or tissue distant from tumor zones vs tissue derived from epileptic foci), composition of cutting solution (NMDG-based vs. sucrose-based), the integrity of the dendritic tree after slicing process and levels of expression of HCN channels.

Given the reviewer’s timely concern about classifying pyramidal neurons as ET and IT-2, and the differences in the magnitude of classic electrophysiological hallmarks (input resistance and sag ratio), we added a section in discussion section addressing this point. In this section, we highlight the electrophysiological features that distinguish ET from IT-2 neurons and discuss possible explanations—such as characteristics related to tissue health or methodological differences—for the variations in input resistance and HCN-mediated sag. This section appears on pages 28-29.

• Rheobase.

Figures 2F and 2I appear inconsistent regarding rheobase. In Fig. 2I, the fitted curves suggest the smallest rheobase in parietal, not frontal, cortex. Fits in Fig. 2I should correspond to measured rheobases in Fig. 2F; please reconcile this mismatch, as it affects a main conclusion.

R: Thanks for pointing out this apparent inconsistency between Figures 2F and 2I. The discrepancy arises from two methods used to estimate or infer rheobase. In Fig 2F, the rheobase was measured using a slow depolarizing ramp protocol that reproduces gradual membrane depolarization and therefore engages processes such as slow Na⁺ inactivation and activation of slow K⁺ currents. In contrast, the curves in Figure 2I were obtained with abrupt depolarizing square current pulses, which favor immediate Na⁺ channel activation and reduce the impact of slow inactivation and accommodation. Because these two protocols probe excitability under different temporal regimes, the inferred (extrapolated) rheobase from F–I data does not necessarily equal the experimentally measured ramp rheobase. For this work, the rheobase was calculated using a depolarizing ramp protocol.

• “Loss of STD”.

The statement “These data suggest that frequency-dependent synaptic facilitation or the loss of STD in epileptic neocortex favors excitatory synaptic activity.” is not supportable without a proper control to link these changes to disease. Therefore, the conclusion should be reframed to avoid implying pathological loss without a proper control. Nevertheless, as I understand the difficulty in getting control tissue, therefore, it is important to underline that the results themselves are valuable.

R: Thanks for this very appropriate comment. Although synaptic depression in response to high-frequency stimulation is well documented under physiological conditions in rodents and near-physiological conditions in the human cortex, we were unable to confirm it due to the absence of control tissue. Therefore, following the reviewer’s suggestion, we have used carefully the term “loss of STD” and instead refer to this observation as a synaptic facilitation in response to high-frequency stimulation. Given that our results indirectly suggest a possible loss of STD, we limited our interpretation to noting it as a lack of STD, which is a more appropriate term and is supported by previous studies.

• Morphology sample size.

Only four biocytin-filled RS PCs (from parietal and frontal cortex) were analyzed. Given known variability with depth, cortical folding, and location within a slice, even within a small region, this is insufficient for strong conclusions. The authors already considered this issue for reviewers #1 and #2, but unfortunately, the issue still stands. Namely, the similarity between the cells could be either statistically significant or not.

R: We acknowledge this limitation of our study. Our intention in retaining these data is to illustrate that a subset of electrophysiologically characterized pyramidal cells was also morphologically identified as LV pyramidal neurons. Ultimately, we aim to make these data accessible to potential readers, given the limited availability of studies combining electrophysiological and morphological measurements in human tissue, and to contribute them, as much as possible, to public datasets such as the Allen Institute Brain Atlas. We have carefully reviewed our manuscript to temper the interpretation of the morphological data and now explicitly mention this as a limitation in the Discussion section.

Important points

• External Ca²⁺.

Because [Ca²⁺]o critically sets release probability, it can strongly alter short-term plasticity. Please, either provide a control demonstrating that your [Ca²⁺]o choice does not change the qualitative outcome, or discuss how different [Ca²⁺] o would impact interpretation.

Thanks for this nice observation. As with all high-probability release synapses, short-term plasticity at cortical LI/II–LV synapses is influenced by the external calcium concentration, which promotes short-term depression as calcium levels increase. Previous studies have shown that calcium concentrations in the range of 1.3–2 mM reliably elicit short-term depression in human cortical synapses from non-epileptic tissue (Testa-Silva et al., 2014; Campagnola et al., 2022). In our study, we used 2.5 mM calcium for short-term plasticity experiments; at this concentration, we would not expect a reversal in short-term plasticity polarity (i.e., from depression to facilitation). In contrast, if the calcium concentration were lower than the physiological range (around 1 mM), the polarity of short-term plasticity would be expected to shift from depression to facilitation, as occurs in high-release-probability synapses, where reduced external calcium induces facilitation due to limited release probability. We have also added a brief discussion in the manuscript addressing the role of the calcium concentration used and the importance of experimental validation in epileptic human tissue. It reads on pages 33-34 as follows:

*It is noteworthy that, although calcium concentration strongly modulates short-term plasticity, in high-probability release synapses, an increase in calcium enhances STD, whereas a reduction favors facilitation (Dittman et al., 2000; Oleskevich et al., 2000;Muller et al., 2008). In our experiments, we used 2.5 mM Ca²⁺, which is a close to the range (1.3-2 mM) that has been shown to reliably elicit STD in cortical synapses from non-epileptic human tissue (Testa-Silva et al., 2014; Campagnola et al., 2022). Therefore, it is unlikely that the synaptic facilitation observed in response to brief 30 Hz train stimulation can be attributed to the calcium concentration used. Nevertheless, this observation warrants corroboration by future studies.*

• “Barely exhibited SSA at RMP.”

If PCs “barely exhibited SSA at their RMP,” what are the consequences for analyses that use SSA as a readout? Please clarify how SSA was ultimately used in Figure 4G.

R: As mentioned in response to the first comment, Figure 4, specifically panels 4C–4F, summarizes the spontaneous postsynaptic potentials recorded using extracellular recordings in response to a 30 Hz stimulation train. We have restricted the term “SSA” to the results from whole-cell recordings, while “spontaneous postsynaptic potentials” refers to recorded events with extracellular recordings. The spontaneous postsynaptic potentials emerged following the 30 Hz stimulation and were classified as such because they occurred ≥10 ms after each stimulus, displayed an inward-directed waveform, had an amplitude >0.15 mV, and a duration >1 ms.

We have updated the y-axis of Figure 4E and the x-axis of Figure 4F to display the number of spontaneous postsynaptic potentials rather than spontaneous events, to avoid confusion with SSA measured using whole-cell recordings. Importantly, Figure 4F shows a positive correlation between the magnitude of synaptic facilitation at the end of the 30 Hz train and the number of spontaneous postsynaptic potentials, suggesting that synaptic facilitation during the 30 Hz train enhances excitatory synaptic drive.

• Rheobase vs threshold.

If rheobase differs across regions while RMP and Rin are similar, this implies a lower voltage threshold for AP initiation in the frontal cortex (≈ 60% closer to rest than the temporal). Can this be confirmed directly from existing data?

R: We measured the threshold at rheobase and confirmed the reviewer’s well-reasoned prediction. This observation has been added to panel J2 of Figure 2, and the results are mentioned on page 22, along with the related methods on page 11 (lines 206-208).

This information is read on page 22 (lines 490-494) from results section as follows:

*Consistent with their lower rheobase and the absence of differences in intrinsic membrane properties, frontal IT-2 neurons exhibited a lower firing threshold compared to temporal neurons* (threshold in frontal: -42.38 ± 5.81 mV; temporal: -32.52 ± 7.61 mV; parietal: -41.12 ± 3.11 mV; one-way ANOVA, F_(2, 19)_ = 5.659, followed by Tukey’s test, *p* < 0.05; right panel in Fig 2J2).

• “Higher global Na⁺ conductance in frontal PCs.”

Could differences in Na⁺ channel kinetics or subtype expression also explain the observations, beyond “global conductance”? A more nuanced report on the observation would help.

R: Yes, that is entirely possible. In the revised version of our manuscript, we have modified a paragraph discussing the potential biophysical properties of voltage-gated Na⁺ channels that may underlie the differences in Na⁺ conductances across cortical regions. Furthermore, we have added references supporting the existence of regional variations in the properties of action potentials and frequency firing among neurons located within the same cortical layer. This modified paragraph reads in discussion on pages 30-31 as follows:

*Nevertheless, the higher excitability observed in frontal IT-2 neurons compared to parietal and temporal neurons may be explained by differences in the biophysical properties of rapidly activating Na⁺ channels rather than by changes in passive properties. For instance, while our measurements of MDS, also referred to as the rise speed of the AP, suggest a higher global Na⁺ conductance in frontal neurons, this measurement may also be influenced by biophysical properties of Na⁺ channels such as voltage dependence, channel density, maximal conductance (Wilbers et al., 2023), and the subunit composition of the channels (Katz et al., 2018). Despite these considerations, previous studies in mice and macaques have reported regional differences in the properties of AP and the firing frequency of layer 5 pyramidal neurons (Miller et al., 2008; Dembrow et al., 2024). Therefore, it is possible that the observed regional differences in excitability are related to the functional specialization of each cortical region (e.g., executive functions in the frontal cortex or sensory processing in the parietal lobe).*

AP dV/dt variance.

Reported slopes (e.g., −83.9 ± 37.1 mV/ms temporal; −48.2 ± 1.96 mV/ms parietal; −77.5 ± 27.4 mV/ms frontal) show a strikingly smaller SD in parietal neurons. Please explain this disparity.

R: This is an interesting observation. First, we checked this data, which corroborated that the calculation was correct. Although we did not aim to specifically examine this difference, a more probable explanation could be the lower variability in patient age for parietal cortex samples (2–7 years) compared to temporal (21–27 years) and frontal (2.6–30 years) cortex samples. Similarly, the duration of epilepsy may influence the variability of action potential kinetics. In this context, parietal neurons were obtained from patients with a shorter duration of epilepsy, which could contribute to more homogeneous electrophysiological properties.

• Human vs rodent slope comparison.

The statement that slopes differ significantly from the rodent control and therefore indicate “altered synaptic response dynamics” is hard to interpret. Why would human microcircuits be expected to match rodent slopes, given anatomical differences (layers and size proportions of the different layers)? Please justify or reframe.

R: We apologize for the lack of clarity in our previous statement. We have carefully revised this section to improve precision and rationale. Specifically, we now justify the relevance of analyzing the relationship between fiber volley amplitude and fEPSP magnitude as a measure of synaptic coupling in neuronal microcircuits (see page 23). This explanation provides context for the subsequent correlation and regression analyses.

In the revised manuscript, we clarify that the slopes obtained from the linear regression between FV and fEPSP slope were used to quantify the magnitude of synaptic coupling (rather than “synaptic gain,” as previously stated). We have also cited studies demonstrating that the magnitude of this relationship’s slope reflects the efficiency of synaptic coupling (page 24). To the best of our knowledge, this is the first time this approach has been applied to human brain slices; however, the theoretical basis remains the same: under physiological conditions, the magnitude of the fEPSP is expected to scale proportionally with the magnitude of the FV, reflecting preserved coupling principles across species. Finally, we have revised the conclusion to replace the phrase “…indicating altered synaptic response dynamics” with “supporting the idea of reduced synaptic coupling across cortical regions in patients with DRE,” which more accurately reflects the data and avoids overinterpretation.

• Use of predicted values.

In figure 4, the authors plot the FV-fEPSP predicted values (from the fitted line). I have to admit that understanding the reasoning for this plotting is not easy. Please consider a clarification for this panel not to burden the reader

We agree with the reviewer that the reasoning of the least-squares methods in our plots is not straightforward and requires statistical justification. The least-squares method is a mathematical procedure that identifies the best-fit line for the observed data and reduces variance, thereby enabling more accurate estimation of the FV-fEPSP relationship. When applied to our data, it yielded linear fits in the temporal, parietal, and frontal cortices, as well as in the control group, consistent with previous reports on the linearity of this relationship (Barnes and McNaughton, 1980; *J. Physiol. 309: 473-85.* Villanueva-Castillo et al., 2017; *Neurobiol. Aging 49: 119-137.* Barón-Mendoza et al., 2025. *Sci Rep. 15(1):749*).

The variance reduction observed in our Pearson correlation plots allowed reliable slope calculations for each experimental group. In general, comparing these slopes allows visualization of deviations from the control condition that may reflect alterations in synaptic transmission efficiency: a steeper slope indicates a more efficient, tightly coupled synapse (Barnes and McNaughton, 1980), whereas a shallower slope suggests partial decoupling, whether due to decreased neurotransmitter release or altered postsynaptic responsiveness.

In our data, however, the FV–EPSP curves exhibited similar slopes across the control and the experimental groups. This indicates that the relationship between presynaptic activation and postsynaptic response remains uniform across all analyzed brain regions. In other words, these similarities suggest that each group preserves a comparable level of synaptic efficiency at a global scale, even if specific local or subregional mechanisms may be differentially affected. This stability reinforces the idea that, despite potential pathological alterations, the fundamental coupling between pre- and postsynaptic activity remains relatively preserved.

• AIS identification.

“The axon’s initial segment was visually identified.” How was the end of the AIS determined? Please reference standard criteria for AIS identification.

R: Thanks for this comment. In our study, the AIS was visually identified as the thin axonal process emerging from the basal region of the soma. The end of the AIS was morphologically defined as the point at which the axon’s diameter became constant, showing no further tapering or structural specialization, and where the axon adopted a uniform caliber and trajectory without visible varicosities or branching. This morphometric criterion is consistent with previous reconstructions of human and rodent cortical neurons, in which changes in axonal diameter and geometry determine AIS length and the transition point (Mohan et al., 2015, *Cerebral Cortex* 25: 4839–4853; Jenkins & Bender, 2025, *Physiological Reviews*, p. 765; Fujitani et al., 2022, *Frontiers in Cellular Neuroscience*, p. 2). Reported AIS lengths in human neocortical pyramidal neurons typically range from 10 to 60 µm (Jenkins & Bender, 2025), which is consistent with our measurements (50 ± 15 µm from the soma). In the revised version of our manuscript, we have clarified in the Methods section the criterion used for AIS identification and provided the appropriate references. The change reads as follows (lines 319-322):

*The axon’s initial segment was identified morphologically as the point at which the axon’s diameter became constant, lacking further tapering or structural specialization, and where the axon exhibited a uniform caliber and trajectory without visible varicosities or branching, as previously reported (Mohan et al., 2015; Jenkins & Bender, 2025).*

**Minor points**

• The Introduction mentions “different neuronal mechanisms at different organizational levels.” Please define these levels explicitly.

R: We have briefly elaborated on this idea regarding potential mechanisms operating at different organizational levels that may contribute to hyperexcitability in the Introduction section. This paragraph (highlighted text) reads as follows:

*Conceptually, multiple neuronal mechanisms operating at different organizational levels may contribute to the emergence of network hyperexcitability. At lower levels, such as the cellular and synaptic, alterations in ion channel function and intrinsic membrane properties can modify cellular excitability. In contrast, changes in synaptic interactions within local circuits can alter synaptic drive and plasticity (Farrell et al., 2019; Stöber et al., 2023), thereby influencing the propagation of electrical activity. At higher levels, including the network and system levels, interactions between neural circuits, or even between the nervous and immune systems, may further promote hyperexcitability (Stöber et al., 2023). Despite significant advances, the precise nature of hyperexcitability remains unknown, raising the question of whether network hyperexcitability in epilepsy results from the convergence of multiple mechanisms across organizational levels or from alterations at only a subset of them.*

• “Glutamatergic strength, the synchronicity between presynaptic volleys and field EPSPs, and short-term, frequency-dependent plasticity were determined at the synaptic level.” Please clarify this phrasing; synaptic properties are, by definition, at the synaptic level.

R: This statement was presented in abstract. To avoid redundancy, we have eliminated the phrase “at the synaptic level” from the mentioned statement.

• “To explore a possible desynchronization… FV vs fEPSP slopes were correlated.” How is “desynchronization” defined here? Do you mean reduced coupling or statistical independence between the two signals?

R: Thanks for this insightful comment. After a careful review of the seminal work by Barnes et al. (1979), we believe that synaptic coupling is a more appropriate term to describe the correspondence between the presynaptic excitability (quantified as the FV) and the postsynaptic responses (fEPSP), which reflects the linear input–output relationship under physiological conditions. Therefore, throughout the manuscript, we have replaced the terms synchronization and desynchronization with coupling and decoupling, respectively, to more accurately reflect the loss of this linear relationship in pathological conditions.

• “≈ 65% of the recorded neurons belong to the IT-2 neurons…” Consider: “≈ 65% of recorded neurons belong to the IT-2 class/ were IT-2.”

R: We have considered this suggestion and now use the phrase: “≈65% of recorded neurons belong to the IT-2 class, providing a more precise understanding. Thank for this observation.

• “Extracellular recordings in independent slices.” Please define “independent” (e.g., different patients, different blocks, or non-overlapping sites?).

R: We apologize for the confusion. We have clarified this point, and the revised sentence now reads (lines 431-433 on page 23) as follows:

*In parallel with the patch-clamp experiments, we also performed extracellular recordings in independent slices obtained from the same patients’ brain samples to explore the synaptic properties of human neocortex.*

• “Strong linear correlation and slope recovery… despite disorganized or desynchronized conditions.” The terms “disorganized” and “desynchronized” are not clear in this context. Please clarify, define precisely, or revise.

R: As mentioned in a previous response, we have replaced the term “desynchronized” with “decoupled” and adjusted similar terms accordingly. Specifically, decoupling refers to the loss of the linear or proportional relationship between FV and fEPSP slope used to estimate input-output coupling in cortical synapses. Likewise, we have removed the term “disorganized” to avoid potential ambiguity or confusion.
